# Improving obesity and lipid metabolism using conjugated linoleic acid

**DOI:** 10.1002/vms3.921

**Published:** 2022-09-14

**Authors:** Ye Sun, Xufeng Hou, Lingjie Li, Yanqing Tang, Mingyue Zheng, Weisen Zeng, XiaoLong Lei

**Affiliations:** ^1^ Department of General Practice Zhujiang Hospital Southern Medical University Guangzhou China; ^2^ Department of Cell Biology School of Basic Medicine Southern Medical University Guangzhou China; ^3^ Department of Nutrition Nanfang Hospital Southern Medical University Guangzhou China

**Keywords:** lipid metabolism, nutrition, obesity

## Abstract

**Background:**

Conjugated linoleic acid (CLA) can prevent fatty acid accumulation induced by a high‐fructose diet and improve lipid metabolism disorders in patients.

**Objectives:**

We aimed to investigate the effect of CLA on obesity and lipid metabolism and its possible mechanism.

**Methods:**

Eight‐month‐old male BKS.Cg‐Dock7^m^ +/+ Lepr^db^/JNju (db/db) mice (*n* = 12) were fed a CLA mix composed of equivalent c9, t11‐CLA and t10, c12‐CLA for 1 month. The effect of CLA on body weight, water and food intake, and triglyceride (TG) and total cholesterol (TC) levels was investigated. PPARα, PPARγ and CD36 expression was determined by quantitative PCR and western blotting. Additionally, the expression of these three genes was studied in HepG2 cells treated with CLA and linoleic acid.

**Results:**

CLA treatment notably reduced the dietary and water intake of db/db mice, effectively reduced body weight, and decreased serum TG and TC levels (*p* < 0.05). Increased expression of PPARα (*p* < 0.05) and decreased expression of CD36 (*p* < 0.001) were observed in the liver of mice that were fed CLA. CLA increased PPARα expression (*p* < 0.001) and decreased PPARγ (*p* < 0.001) and CD36 expression (*p* < 0.01) in HepG2 cells.

**Conclusions:**

Our results showed that CLA can improve lipid metabolism in obese mice through upregulation of PPARα expression and downregulation of CD36 expression.

## Graphical abstract

CLA can prevent obesity and improve lipid metabolism in mice. CLA significantly decreased the body weight and reduced the serum levels of TG and TC. CLA might be involved in controlling fatty acid uptake by regulating the expression of PPARα and CD36.

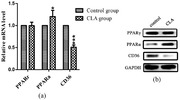

## 1 INTRODUCTION

With development of the economy and improvement of living standards, the incidence rate of obesity worldwide has increased rapidly. Obesity severely harms human health as it can cause fatty liver, type 2 diabetes and malignant tumours. Simultaneously, abnormal lipid metabolism has become a major health problem worldwide.

Conjugated linoleic acid (CLA) comprises several positional and geometric isomers of linoleic acid (LA; C18:2, *n*‐6) of which c9, t11‐CLA (90%) and t10, c12‐CLA (10%) isomers occupy the main proportion (Pariza & Ha, 1990a,b). CLA isomers actively inhibit carcinogenesis and prevent atherosclerosis, diabetes, obesity, tumours and osteoporosis, as well as play a role in autoxidation and the immune system (Pariza et al., 1979; Pariza & Hargraves, 1985; Banu et al., 2008; Belury, 2002; Choi & Seo, 2013; Chinnadurai et al., 2013; Viladomiu et al., 2016). Recently, the FDA has approved CLA as ‘Generally Recognised as Safe’; therefore, it can be used in various food and beverage items. CLAs can prevent fatty acid accumulation induced by a high‐fructose diet, indicating that CLA can improve lipid metabolism disorders in patients (Maslak et al., 2015).

In this study, we investigated the effect of CLA on the expression of lipid metabolism‐associated genes in db/db mice.

## 2 MATERIALS AND METHODS

### 2.1 Animals and diets

Twelve 8‐month‐old male BKS.Cg‐Dock7^m^ +/+ Lepr^db^/JNju (db/db) mice were purchased from Jackson Laboratories (Bar Harbor, Maine 04609 USA). Weight‐matched mice were randomly divided into the following two groups: CLA and control. The CLA group was fed 200 µl CLA mix (Qingdao AoHai biological Co. LTD) daily, and the control group was fed 200 µl saline solution at the same time. They were all fed a standard diet and maintained in a temperature‐controlled room (22‐25°C, 45% humidity) on a 12 h light‐dark cycle. Body weight and food and water intake were measured every 3 days. Animal experiments strictly followed the National Institutes of Health guidelines and were approved by the Institutional Laboratory Animal Care and Use Committee of the South Medical University at Guangzhou (Approval No. L2017104, Guangzhou, Guangdong province, China) (Deng et al., 2019).

### 2.2 Blood Biochemistry

At the end of the experiment, blood was collected from the heart after anaesthesia. Next, the serum was collected and total cholesterol (TC) and total triglycerides (TG) were measured using an automated biochemical analyser (HITACHI 7150, Japan).

### 2.3 HepG2 cell treatment

HepG2 cells were treated with c9, t11‐CLA and t10, c12‐CLA (Cayman Chemical Company, USA) at a concentration of 50 µl in the CLA group. The negative control group was treated with LA (Macklin, Shanghai, China).

### 2.4 Quantitative reverse‐transcription (qRT)‐PCR

Total RNA was extracted from liver tissues or HepG2 cells using the Trizol method and then quantified using a spectrophotometer (NanoDrop 1000, NanoDrop Technologies, Wilmington, DE, USA). The expression of CD36, PPARγ and PPARα was measured by qRT‐PCR using the following primers: 5′‐AAGCTATTGCGACATGATT‐3′ (forward), 5′‐GAT CCG AAC ACA GCG TAG AT‐3′ (reverse); 5′‐CGAGAAGGAGAAGCTGTTGG‐3’(forward), 5′‐TCA GCG GGA AGG ACTTTATG‐3′ (reverse); and 5′‐CTCCCTCCTTACCCTTGGAG‐3′ (forward), 5′‐GCC TCT GAT CACCACCATTT‐3′ (reverse), respectively. GAPDH expression was monitored using the primers 5′‐GGACCTCATGGCCTACATGG‐3′ (forward) and 5′‐TAG GGC CTC TCT TGC TCA GT‐3′ (reverse).

### 2.5 Western blotting

Total protein was extracted from liver tissues or HepG2 cells. Samples were separated using sodium dodecyl sulphate‐polyacrylamide gel electrophoresis and electroblotted onto polyvinylidene difluoride membranes. The membranes were first blocked with non‐fat milk for 2 h, and incubated with the following primary antibodies: anti‐PPARγ, anti‐PPARα, anti‐CD36(ABclonal Technology, Boston, MA, USA), anti‐GAPDH and anti‐α‐tubulin (Beijing Ray antibody, Beijing, China), followed by incubation with a horseradish peroxidase‐conjugated anti‐rabbit or mice secondary antibody (Beijing Ray antibody). Objective proteins were detected using ECL Plus (Pierce, NJ, USA).

### 2.6 siRNA interference and immunofluorescence

The sequence of interference RNA for gene silencing was as follows: PPARα sense 5′‐ggcacuaacaccuct‐3′, anti‐sense 5′‐agguggauguacucucucucucucucucct‐3′; and PPARγ sense 5′‐ccaaguguaguuguuugcuudtdt‐3′, anti‐sense 5′‐acaacagaacucuaaaacuggdtdt‐3′.

The cells were inoculated into a six‐well plate, and 3.75 µl of Lipo3000(ThermoFisher Scientific, USA) and 40 pmol of siRNA (Shanghai Jima Gene Company, Shanghai, China) were added to each well. To dilute the siRNA, optiMEM (125 µl) and lipo3000 transfection reagent were used. After incubating for 48 h, the RNA was extracted for the fluorescence quantitative PCR experiment. The protein was extracted for 48–72 h by western blotting.

Bodipy^TM^ FL C16(Thermo Fisher Scientific) was diluted with dimethyl sulphoxide, 20 µmol/L was added into each well, and then cultured with the transfection reagent for 48 h. After treatment, the cells were observed using a laser confocal microscope.

### 2.7 Statistical analysis

Results are shown as the mean ± standard error of the mean. Based on the data obtained, a two‐way analysis of variance, Kruskal‐Wallis or Fisher exact test was performed. *p* < 0.05 was considered statistically significant. All data were analysed using the Statistical Program for Social Sciences version 21.0.

## 3 RESULTS

### 3.1 Food and water intake and body weights

**FIGURE 1:**
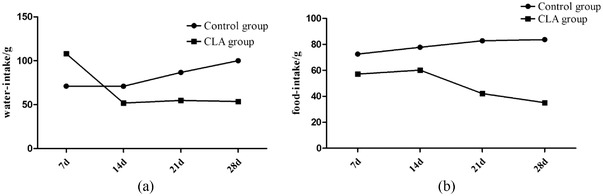
Water and food intake amount of the control and CLA groups. (a) Water intake amount (g). (b) Food intake amount (g)

**FIGURE 2:**
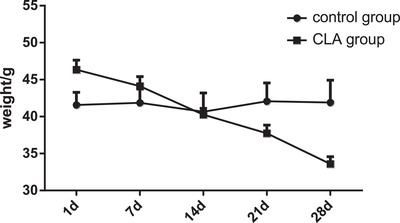
Body weight changes (g) in the control and CLA groups.

After 1 month of treatment, the water and food intake markedly decreased in the CLA group compared with that in the control group (Figure 1a and b). Furthermore, the body weight decreased to normal after treatment with CLA (Figure 2).

### 3.2 TC and TG levels

**FIGURE 3:**
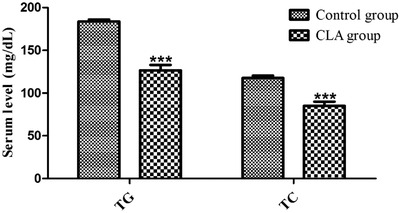
Serum TG and TC levels in the control and CLA groups. ***p < 0.001

The TC and TG levels were markedly lower (*p* < 0.001) in the CLA group than in the control group (Figure 3).

### 3.3 Gene expression analyses (quantitative PCR)

**FIGURE 4:**
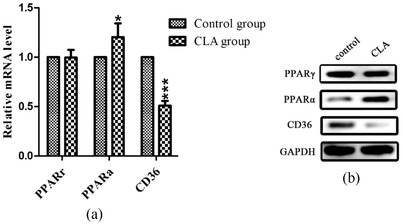
Comparison of expression levels of PPARγ, PPARɑ and CD36 genes in the control and CLA groups. (a) Detection of mRNA expression of the three genes in the two groups qRT‐PCR. (b) Determination of protein expression quantity of the three genes in the two groups by western blotting. *p < 0.05, ***p < 0.001

**FIGURE 5:**
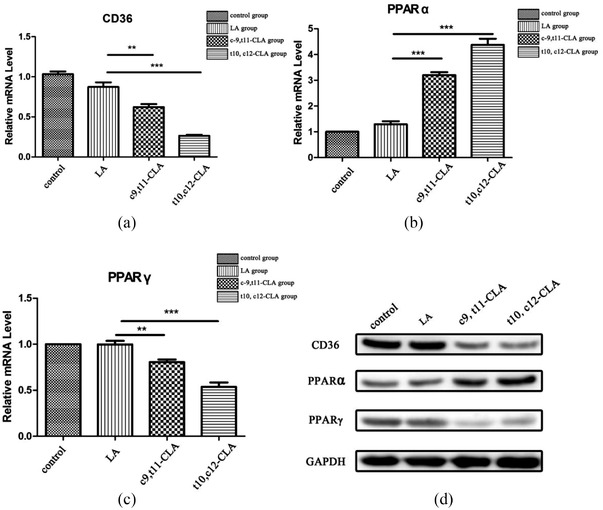
mRNA and protein expression levels of CD36, PPARα and PPARγ after treatment with linoleic acid, c‐9, t11‐CLA, or t10, c12‐CLA. (a)–(c) qRT‐PCR results. (d) Western blotting results. **p < 0.01, ***p < 0.001

In the in vivo experiments, the CLA mix treatment considerably decreased the expression of CD36 in the liver compared with that in the control group and showed a tendency to increase the expression of PPARα (Figure 4a). In the in vitro experiments, after the treatment of HepG2 cells with CLA for 48 h, RNA was extracted for fluorescence quantitative PCR detection. The results showed that after treatment with CLA, the mRNA expression of the PPARγ gene decreased significantly (Figure 5a), while that of the PPARα gene increased (Figure 5b) and the CD36 gene decreased (Figure 5c).

### 3.4 Western blotting

The expression level of each protein obtained from the in vivo experiments was ascertained (Figure 4b). PPARα expression increased, while CD36 expression decreased, in the CLA group compared with that in the control group. The results were in accordance with those of the qRT‐PCR analysis. The in vitro experimental results were slightly different from those of the in vivo experiments, as the expression of PPARα increased, while that of CD36 and PPARγ decreased, in the in vitro experiments (Figure 5d).

### 3.5 siRNA interference and immunofluorescence

**FIGURE 6:**
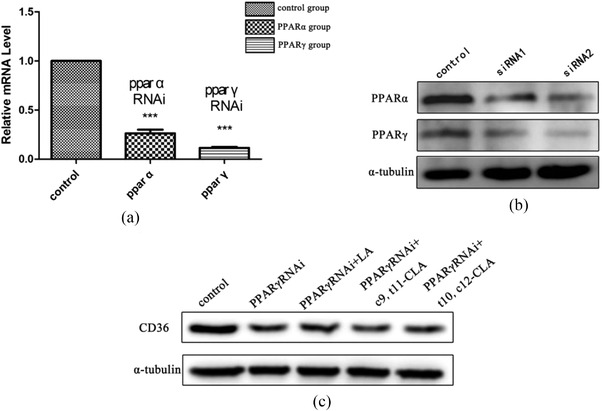
(a) mRNA expression level of PPARγ is significantly decreased after gene knockdown. (b) The protein expression level of PPARγ is significantly decreased after gene knockdown. (c) CD36 protein expression is significantly decreased after PPARγ gene knockdown. ***p < 0.001

**FIGURE 7:**
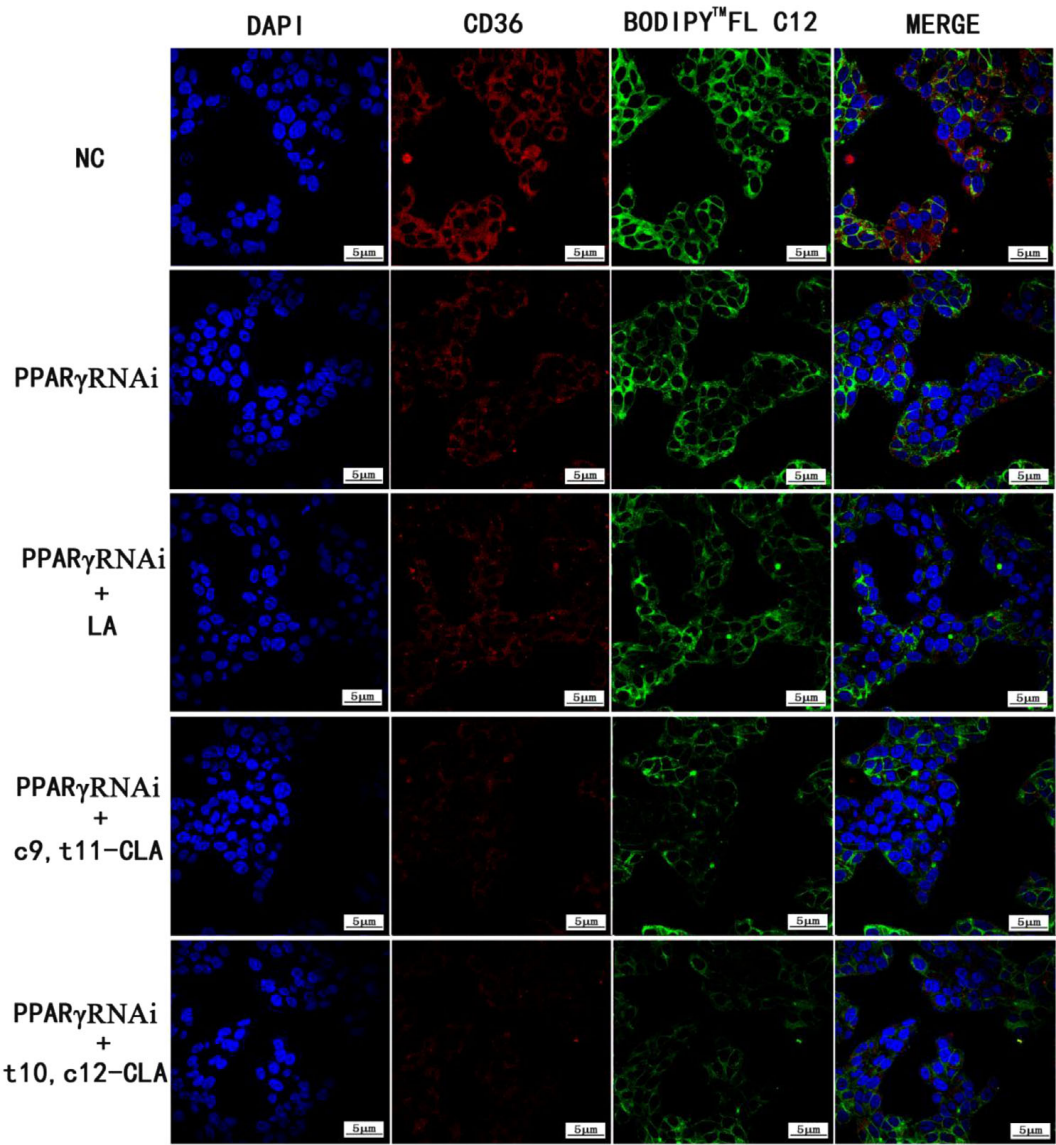
Expression of BODIPY^TM^ FL C16, PPARαRNAi and CD36 using a laser confocal microscope (600×)

After siRNA transfection, PPARγ and CD36 protein expression decreased significantly in the HepG2 cells (Figure 6a–c). The PPARγ knockout group showed weaker green fluorescence signal (CholEsteryl Bodipy^TM^ FL C12) and red fluorescence signal (CD36) than the control group. However, the CLA group showed weaker green and red fluorescence signals than the PPARγ knockout group (Figure 7).

## 4 DISCUSSION

Dietary CLA can markedly reduce body fat mass (Hayman et al., 2002; Blankson et al., 2000). CLA comprises several positional and geometric isomers of which c9 t11‐CLA and t10 c12‐CLA are the main components. In this study, db/db mice were fed a CLA mix, and our results showed that CLA can significantly reduce body weight and improve TG and TC levels. Previous studies have also shown that CLA mix can significantly decrease body weight and fat mass (Park et al., 1997; Park et al., 1999; West et al., 1998). In our study, the CLA supplement markedly affected food and water intake in addition to body weight; these findings were consistent with those of prior investigations (Park et al., 1999; Hargrave et al., 2004).

Furthermore, to elucidate the mechanism underlying the effects of CLA on lipid metabolism, we investigated the expression of three genes associated with lipid metabolism: PPARγ, PPARα and CD36. The qRT‐PCR and western blotting results showed that CD36 expression significantly decreased, while that of PPARα increased in the CLA group. PPARα is highly expressed in mitochondria‐rich and active β‐oxidation tissues such as the liver, renal cortex, intestinal mucosa and heart. PPARα comprises both natural and synthesised ligands. Natural ligands include polyunsaturated fatty acids (PUFAs), unsaturated fatty acids, and inflammatory mediators, among which PUFAs have the highest affinity. PPARα can promote fatty acid oxidation and ketogenesis, decrease blood glucose and regulate the uptake and storage of lipids (Ferré, 2004). A PPARα ligand has been shown to decrease TG levels and increased high‐density lipoprotein levels (Duez et al., 2005). Similarly, in our study, the increased expression of PPARα might account for the decreased TCnd TG levels. CD36 is a membrane protein that regulates fatty acid uptake in various cell types such as monocytes, platelets, macrophages, microvascular endothelial cells, adipocytes, muscle cells, enterocytes and hepatocytes. Liver cells can take up fatty acids through CD36 (Buqué et al., 2012). Moreover, CD36 levels in the liver are positively correlated with fatty liver disease (Petta et al., 2013; García‐Monzón et al., 2014). In our previous study, we performed fluorescent labelling of fatty acids and demonstrated that PPARγ regulates the absorption of fatty acids through CD36 (Kassis et al., 2012). Recently, enhanced hepatic CD36 expression with age was shown to be associated with enhanced susceptibility to fatty liver in both mice and humans (Sheedfar et al., 2014), which was also consistent with our current results.

In conclusion, we demonstrate that CLA can prevent obesity and improve lipid metabolism in mice. CLA significantly decreased the body weight and reduced the serum levels of TG and TC. Furthermore, we reveal that CLA might be involved in controlling fatty acid uptake by regulating the expression of PPARα and CD36. The detailed mechanism of action of CLA should be investigated in future studies.

## AUTHOR CONTRIBUTIONS

Ye Sun: Data curation; investigation; methodology; project administration; validation; visualisation; writing – original draft; writing – review & editing. Xufeng Hou: Data curation; investigation; validation; visualisation. Lingjie Li: Investigation; visualisation. Yanqing Tang: Visualisation. Mingyue Zheng: Visualisation. Weisen Zeng: Funding acquisition; supervision. Long Xiao Lei: Conceptualisation; funding acquisition; methodology; resources; supervision.

## CONFLICT OF INTEREST

The authors declare no potential conflicts of interests.

### ETHICS STATEMENT

The authors confirm that the ethical policies of the journal, as noted on the journal's author guidelines page, have been adhered to and the appropriate ethical review committee approval has been received. The authors confirm that they have followed EU standards for the protection of animals used for scientific purposes.

### PEER REVIEW

I would not like my name to appear with my report on Publons https://publons.com/publon/10.1002/vms3.921

## Data Availability

The data that support the findings of this study are available on request from the corresponding author. The data are not publicly available due to privacy or ethical restrictions.
